# Antibody-conjugated nanoparticles for target-specific drug delivery of chemotherapeutics

**DOI:** 10.3762/bjnano.14.75

**Published:** 2023-09-04

**Authors:** Mamta Kumari, Amitabha Acharya, Praveen Thaggikuppe Krishnamurthy

**Affiliations:** 1 Department of Pharmacology, JSS College of Pharmacy, JSS Academy of Higher Education & Research, Ooty, The Nilgiris, Tamil Nadu, India; 2 Biotechnology Division, CSIR-Institute of Himalayan Bioresource Technology, Palampur (H.P.) 176061, India; 3 Academy of Scientific & Innovative Research (AcSIR), Ghaziabad-201002, India

**Keywords:** active targeting, chemical conjugation, chemotherapeutics, drug delivery, monoclonal antibody

## Abstract

Nanotechnology provides effective methods for precisely delivering chemotherapeutics to cancer cells, thereby improving efficacy and reducing off-target side effects. The targeted delivery of nanoscale chemotherapeutics is accomplished by two different approaches, namely the exploitation of leaky tumor vasculature (EPR effect) and the surface modification of nanoparticles (NPs) with various tumor-homing peptides, aptamers, oligonucleotides, and monoclonal antibodies (mAbs). Because of higher binding affinity and specificity, mAbs have received a lot of attention for the detection of selective cancer biomarkers and also for the treatment of various types of cancer. Antibody-conjugated nanoparticles (ACNPs) are an effective targeted therapy for the efficient delivery of chemotherapeutics specifically to the targeted cancer cells. ACNPs combine the benefits of NPs and mAbs to provide high drug loads at the tumor site with better selectivity and delivery efficiency. The mAbs on the NP surfaces recognize their specific receptors expressed on the target cells and release the chemotherapeutic agent in a controlled manner. Appropriately designed and synthesized ACNPs are essential to fully realize their therapeutic benefits. In blood stream, ACNPs instantly interact with biological molecules, and a protein corona is formed. Protein corona formation triggers an immune response and affects the targeting ability of the nanoformulation. In this review, we provide recent findings to highlight several antibody conjugation methods such as adsorption, covalent conjugation, and biotin–avidin interaction. This review also provides an overview of the many effects of the protein corona and the theranostic applications of ACNPs for the treatment of cancer.

## Introduction

Off-target side effects, such as myelosuppression, mucositis, alopecia, organ dysfunction, and thrombocytopenia, are the most significant clinical challenge when using conventional chemotherapeutics [1]. To improve therapeutic efficacy and to reduce off-target side effects, strategies such as cancer cell-specific targeted delivery, thermally responsive polymer–drug conjugates, macromolecule drug conjugates, gene-directed enzyme prodrug therapy, small molecule drug conjugates, and others are being investigated [2–3]. Targeted delivery with nanoparticles (NPs) has received a lot of attention because it reduces toxicity while also providing good drug compatibility and loadability. Furthermore, NPs increase drug circulation time and serum stability. Also, they enable drug release in a sustained and controlled manner [4]. Targeted delivery of drug-loaded NPs can be achieved either through passive targeting, where drugs accumulate in tumor tissues via the enhanced permeability and retention (EPR) effect, or through active targeting via the functionalization of ligands, such as antibodies or proteins, that interact with receptors overexpressed at the target site [5–6].

**Figure 1:**
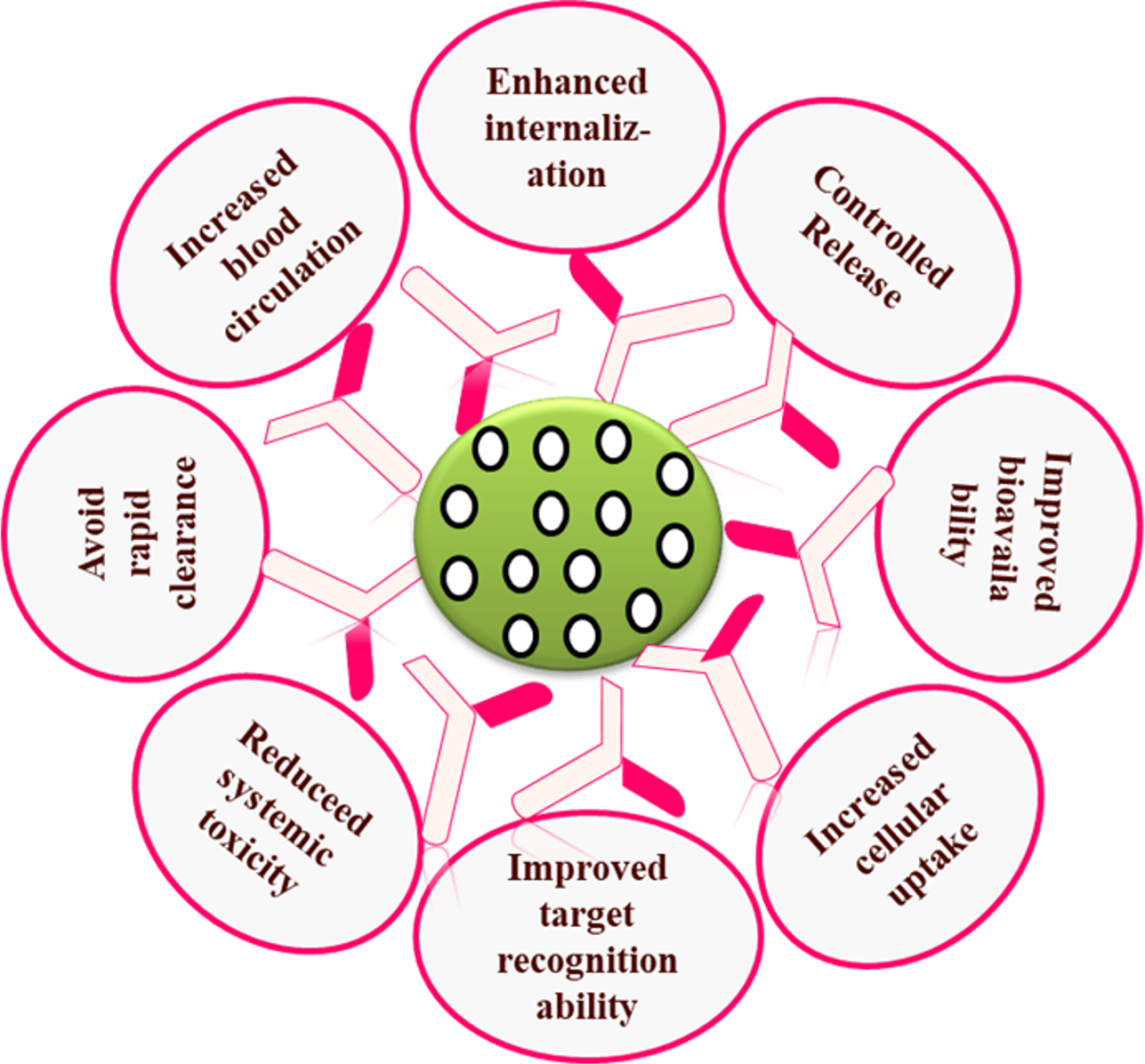
Advantages of antibody-conjugated NPs.

However, the movement of NPs is hampered by biological barriers such as endothelial, cellular, skin, and mucosal barriers, which obstruct their targeting capabilities [7]. Researchers focused their interest on understanding the obstructions that impede targeted drug delivery, and several advances have been made to develop NPs with enhanced ability to cross these barriers. Bio-pharmacological drugs, which include recombinant proteins, monoclonal antibodies (mAbs), and nucleic acid-based materials for targeted drug delivery, have been approved by the Food and Drug Administration (FDA) for the treatment of cancer, arthritis, asthma, psoriasis, pemphigus vulgaris, and chronic urticaria [8]. Antibodies are the primary homing ligands in tumor-targeted drug delivery because of their high specificity, recognition ability, and intracellular stability [9–10]. The mAb-mediated targeted drug delivery specifically eradicates tumor cells without causing systemic toxicity associated with conventional chemotherapeutic agents [11]. Complete mAbs or just the fragment antigen-binding (Fab) region of mAbs are chemically conjugated to NP surfaces to recognize protein targets that are overexpressed on the surface of tumor cells. Conjugation of mAbs to NP surfaces improves targeting capacity, cellular uptake, and intracellular stability [12]. The mAb-functionalized NPs specifically bind to the cell surface proteins and deliver the drug cargo to tumor sites via passive or active targeting. As a result, the therapeutic ratio is improved. At the same time, the systemic toxicity is reduced and the therapeutic efficacy is increased [13]. Antibody-conjugated NPs (ACNPs) combine advantages of NPs and antibodies, which results in more specific and efficient delivery systems (Figure 1).

There are a number of approaches for achieving specific conjugation of antibodies on NP surfaces. The selection of the most suitable conjugation method is very important to preserve antigen binding ability. Improper antibody conjugation influences antigen binding affinity and specificity. Once injected into the body, the ACNPs face both physical and biological barriers (such as diffusion, flow and shear forces, aggregation, protein adsorption, phagocytic sequestration, and clearance), which eventually decrease the number of NPs at the target site [14]. In blood stream, proteins get adsorbed onto the NPs and form a protein corona. The proteins from the biological environment produce a screening effect, which affects the targeting ability of the NPs [15–16]. Protein corona formation on the surface of NPs can also reduce the EPR effect and results in the rapid clearance of NPs from systemic circulation [16]. The focus of this review is to provide an update on commonly used conjugation techniques along with their merits and demerits, as well as the multivalent behavior of antibody-conjugated NPs. In this review, recent studies regarding effects of the protein corona and the theranostic application of ACNPs are highlighted to provide an update of the current research for cancer treatment.

## Review

### Antibodies

**Figure 2:**
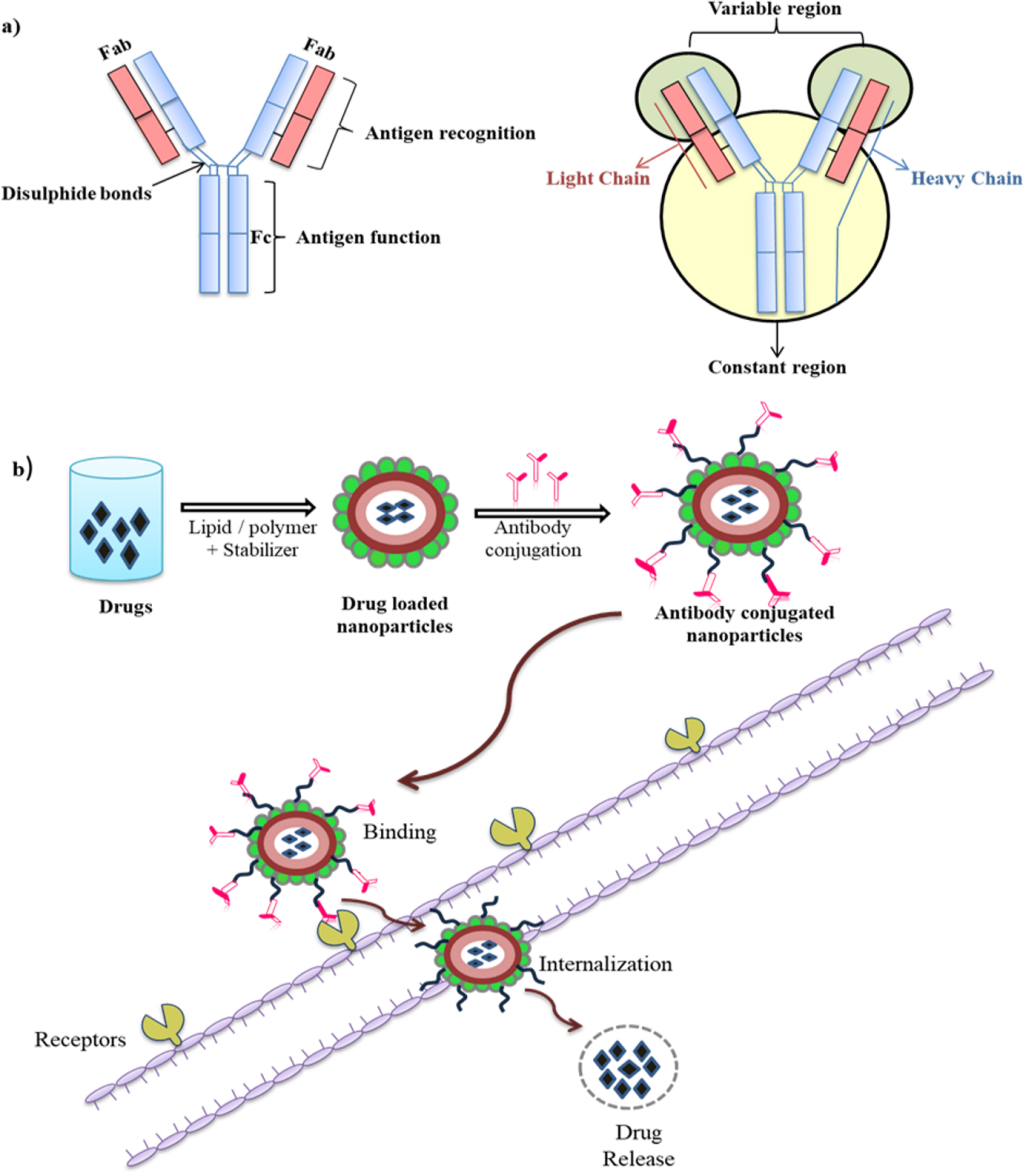
Schematic representation of (a) an antibody and (b) targeted delivery via antibody-conjugated NPs.

Antibodies are Y-shaped glycoproteins produced by B-lymphocytes. These react specifically with antigens, which are responsible for the production or induction of specific antibodies [17]. The specific binding of antigens to their receptors activates a signaling pathway in B-cells, which leads to the secretion of antibodies into biological fluids [18]. Antibodies have four polypeptide chains, that is, two heavy and two light chains (Figure 2a). The two heavy chains are linked together via disulfide bonds. Also, each heavy chain is bonded this way to one light chain. Because of two identical antigen binding sites, the antibody has the ability to bind simultaneously with two identical structures; therefore, antibodies are extensively used for protein recognition and targeting [19]. The mAbs are highly specific and capable to induce selective cellular toxicity by binding with specific target antigens, which results in cell lysis either by antibody-dependent cellular cytotoxicity, complement activation, complement-dependent cytotoxicity, or by inhibition of signal transduction [20–21].

### Molecular cancer targets

Cancer is a highly heterogeneous condition that arises from several mutations in transforming and tumor suppressor genes. High rates of metastasis, invasion, relapse, and drug resistance are the main causes of treatment failures in cancer [22]. Therefore, there is a need of smart delivery systems to eliminate even the last cancerous cell that might lead to tumor reoccurrence. Novel cancer targets have been identified based on the recent understanding of various molecular mechanisms involved in cancer, such as apoptotic proteins (e.g., Bcl-2 survival protein, tumour protein p53, tumour necrosis factor, and nuclear factor kappa-B) [23–24], cancer surface markers (CD44, CD133, and ALDH1) [25] signaling pathways (e.g., PI3K/AKT/mTOR pathway, Hippo pathway, Wnt/β-catenin pathway, JAK2/STAT3 pathway) [25–28], and proangiogenic factors (e.g., vascular endothelial growth factor receptor, epidermal growth factor receptor (EGFR), platelet derived growth factor, and basic fibroblast growth factor) [29–30]. Further, overexpression of cancer receptors, such as estrogen receptor (ER), folate receptors (FRs), human epithelial receptor (HER-2) and transferrin receptors (TfRs) [30–34], has been explored extensively. The selective targeting of these molecular targets via antibody-conjugated NPs provides an efficient platform to accurately deliver the drug cargo specifically to the target site and inhibit the cancer progression.

### Nanoparticle surface decoration strategies

Surface functionalization of NPs through conjugation of functional groups with biomolecules is one method to enhance the targeting efficiency (Figure 2b). For effective antibody functionalization, the involvement of Fab regions is generally avoided during conjugation, so that antibodies do not lose their antigen recognition sites [35]. The antibody immobilization on NP surfaces can be either random or oriented, depending on the functionalization method. Antibodies conjugated onto NPs are able to adopt different spatial orientations due to their asymmetric nature.

Additionally, the functionalization also alters the surface composition and morphology of the NPs. The nature of interactions between NPs and antibodies is directly related to the type of functional groups present on the NPs surface and the surface charge [36–37]. Surface modification strategies include, for example, adsorption, covalent conjugation, and biotin–avidin interaction, which will be discussed below in detail with examples.

#### Adsorption

**Figure 3:**
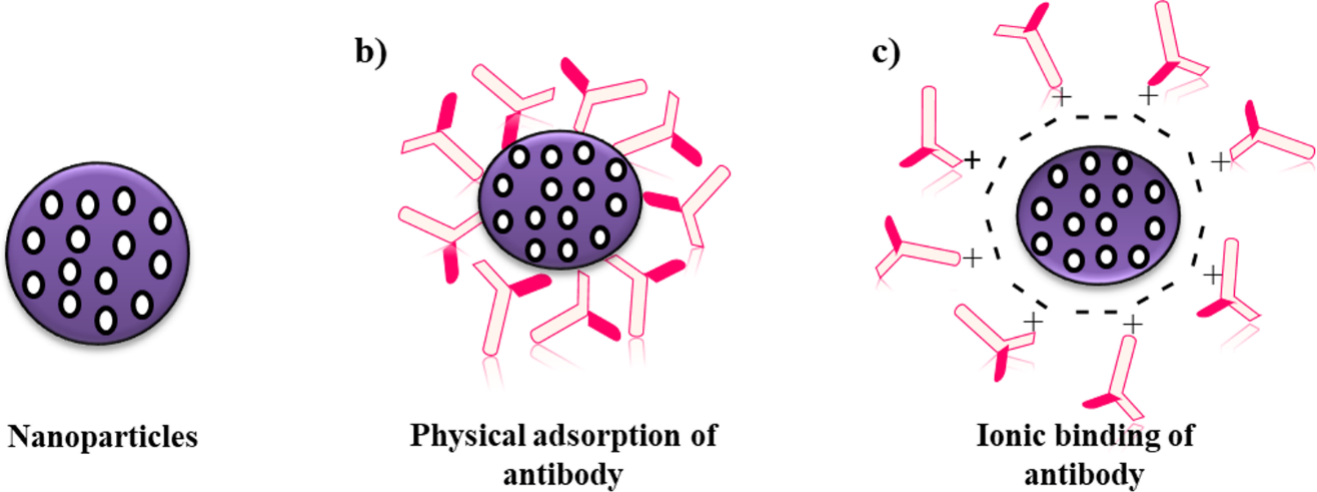
Non-covalent antibody functionalization. (a) Drug-loaded NPs, (b) physical adsorption of antibodies on the nanoparticle surface, and (c) electrostatic binding of antibodies on the nanoparticle surface.

Adsorption of antibodies on the surface of NPs is a non-covalent reversible binding method, which includes physical adsorption and electrostatic binding (Figure 3) [38]. Physical adsorption consists of either non-covalent weak hydrophobic or electrostatic hydrogen bonding, or attractive van der Waals interaction between antibodies and NPs [39].

Ionic binding, in contrast, involves an interaction between surface of the antibody and surface of the NP, which are oppositely charged [40]. This method is widely used for antibody conjugation because it is simple and less time-consuming. Recently, Choi et al. used an adsorption method to coat the surface of docetaxel nanocrystals (DTX-NCs) with Herceptin® to improve cellular uptake and cytotoxicity in breast cancer cells [41]. Similarly, Rayavarapu et al. conjugated HER2 antibodies on the surface of gold nanoparticles using a noncovalent conjugation method in order to increase intracellular uptake into cancer cells [42]. The adsorption results demonstrated that no additional steps for conjugation were required, such as antibody modification or NP surface group activation. However, there are several drawbacks of this method. The method is the least stable and requires high concentrations of antibodies for adsorption. Hydrophobic interaction can also induce conformational changes, which result in denaturation and loss of activity [43]. Similarly, electrostatic interactions between oppositely charged NPs and antibodies result in weak interactions where the antibodies are easily detached due to small changes in pH or ionic strength [44].

#### Covalent binding

Covalent binding of antibodies can be achieved either by adding functional groups on the NP surfaces or by chemical modification of antibodies. Covalent attachment provides high stability, prominent reproducibility, and strong interaction; therefore, changes of pH or ionic strength do not affect the interaction between antibodies and NP surfaces [45–46]. The most commonly used covalent binding techniques are related to the interaction between NPs and antibodies via carbodiimide crosslinker chemistry, maleimide-activated crosslinker chemistry, and click chemistry.

**Figure 4:**
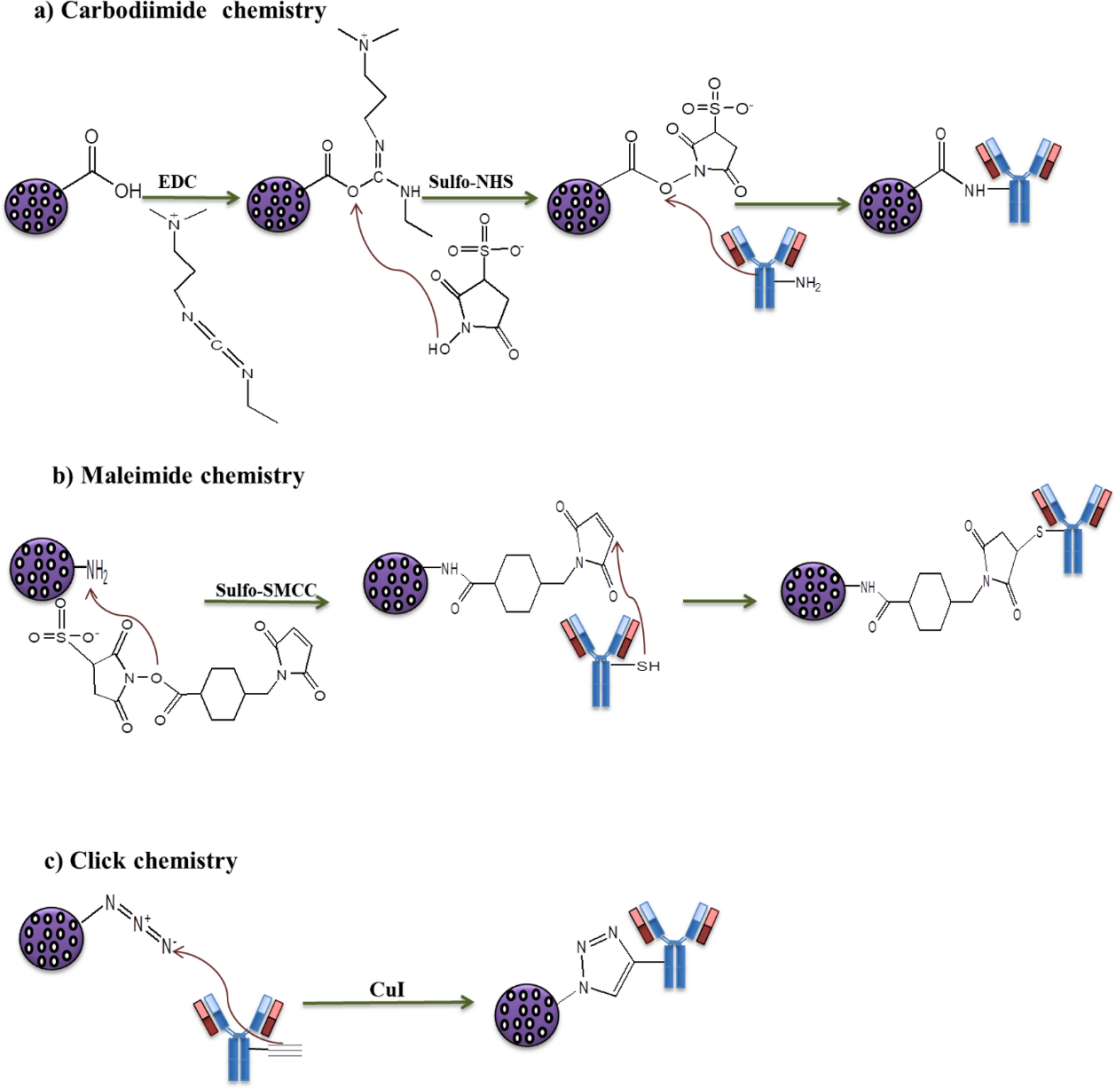
Covalent antibody functionalization techniques. (a) Carbodimide coupling, (b) maleimide coupling, and (c) click coupling of antibodies on nanoparticle surfaces.

**Carbodiimide chemistry:** Carbodiimide conjugation is the commonly used coupling method for covalent binding of antibodies on NP surfaces by primary amine groups. Amine groups are abundant on the antibody surfaces and are highly susceptible for any reaction without chemical modification towards the various functional groups present on NP surfaces, such as aldehyde, carboxylic acid, and epoxide groups [46–47]. The carboxyl groups on the NP surfaces are activated in the presence of 1-ethyl-3-(-3-dimethylaminopropyl) carbodiimide (EDC), and a zero-length carboxyl-to-amine crosslinker forms an amide bond through coupling with amine groups of the antibodies (Figure 4a) [35,47]. Neither *N*-hydroxysuccinimide (NHS) nor its water-soluble analogue (sulfo-NHS) are required for the single-step carbodiimide reaction. However, NHS or sulfo-NHS are generally added to produce dry-stable intermediates, which improves the conjugation efficiency. In a two-step reaction, NHS/sulfo-NHS evades the intra- and intermolecular cross-linking of the antibodies as the antibodies have both amine and carboxyl groups [48]. Acharya et al. prepared rapamycin-loaded polymeric PLGA NPs and conjugated them with cetuximab using EDC/NHS cross-linking chemistry. The antibody-conjugated NPs were able to recognize the extracellular ligand-binding domain of EGFR and provided an effective targeted delivery of rapamycin. The ACNPs significantly increased the therapeutic effect of the chemotherapeutics [49]. The covalent binding can lead to a random immobilization of antibodies on the NP surfaces, because at physiological pH (pH 7), the most reactive amine groups are situated in the Fab region, which further leads to the loss of biological activity [50–51]. To avoid this, other techniques with oriented immobilization are mostly preferred for conjugation.

**Maleimide chemistry:** Maleimide chemistry is another covalent immobilization technique, which comprises binding through thiol groups (–SH) of the antibodies. This oriented covalent conjugation technique requires various steps, such as chemical modification achieved either by oxidation of sugar moieties or the reduction of disulfide bonds. Thiols, also called sulfhydryls, in the cysteine side chain are slightly less abundant than primary amines; therefore, the coupling by thiol groups is more selective [52]. Thiols in cysteines are linked by disulfide bonds (–S–S–) through an oxidation process where the thiols groups are oxidized into disulfides.

The disulfide bonds stabilize tertiary and quaternary protein structures and play a crucial role in protein folding [53–54]. For conjugation, free thiol and reduced thiol groups are required. Many proteins contain cysteine moieties linked with thiol. In proteins that do not have free thiol groups, such groups can be generated either by reducing the disulfide bonds (DTT and BME) or by introducing cysteine residues (Traut’s reagent and *N*-succinimidyl *S*-acetylthioacetate) at different positions. The reaction with primary amines or the reduction of disulfide bonds of the antibodies form free thiol groups. The free thiol groups of the antibody can be linked to the primary amines on NP surfaces using SMCC, sulfo-SMCC, and their analogues via maleimide coupling (Figure 4b). These crosslinkers react specifically with thiol groups at pH 6.5 to 7.5 and form stable irreversible thioether linkages [55]. Swaminathan et al. used the maleimide conjugation technique to coat the surface of paclitaxel-loaded-PLGA NPs with anti-CD133 antibody. The antibody-conjugated NPs improved the intracellular uptake of paclitaxel as well as the targeting effectiveness of the NPs. Furthermore, the antibody-conjugated NPs yielded a highly effective site-specific NP release of the chemotherapeutic agent and an overall increase the therapeutic efficacy [56].

Covalent binding through sugar chains of the antibodies results in the oriented immobilization of antibodies. The sugar moieties in the fragment crystallization (Fc) region get oxidized to form aldehyde groups in the antibody, which react with NPs having amine groups on their surface through reductive amination [57]. For this coupling, having glycosylated antibodies is the major criterium; however, some recombinants or mAbs do not have sugar moieties in their structure.

**Click chemistry:** Click chemistry is characterized as a group of chemical reactions with orthogonality and site-specificity. This chemistry yields promising reaction rates with high efficiency in aqueous solutions and generates minimal cytotoxic byproducts. Click chemistry involves a copper-catalyzed cycloaddition between an organic azide and a terminal alkyne to form a stable 1,4-disubstituted 1,2,3-triazole ring (Figure 4c) [58–59]. Azides and alkynes are inert towards most functional groups and biomolecules. Hence, the NPs are functionalized with these molecules using EDC/NHS and maleimide conjugation techniques for site-specific bioconjugation [60]. This conjugation is highly selective because it does not interfere with organic groups such as amine and carboxyl groups in the antibodies. Additionally, it does not require controlled pH conditions for coupling. Shi et al. developed a polymeric nanoparticle system using biodegradable graft copolymers of (poly(TMCC-*co*-LA)-*g*-PEG-furan) to conjugate anti-HER2 antibodies through a Diels–Alder reaction. They used furan groups (diene), which are accessible for reaction with antibodies functionalized with maleimide (dienophile) groups. The ACNPs increased the cytotoxicity, specificity, and intracellular uptake of the chemotherapeutic in cancer cells and resulted in a better therapeutic efficacy. Accumulating evidence revealed that click chemistry provides site-specific bioconjugation by increasing the antigen binding capacity and the conjugation efficiency [61].

These covalent conjugation methods are highly stable but associated with some drawbacks. These include aggregation and polymerization, cross-linking at multiple sites on the antibodies, the need for additional purification steps for the removal of linkers and catalytic agents, which results in low yield and poor reproducibility, and the random orientation of antibodies on the NP surfaces, resulting in a low accessibility of antigen binding sites [62].

#### Binding by adapter molecules

**Figure 5:**
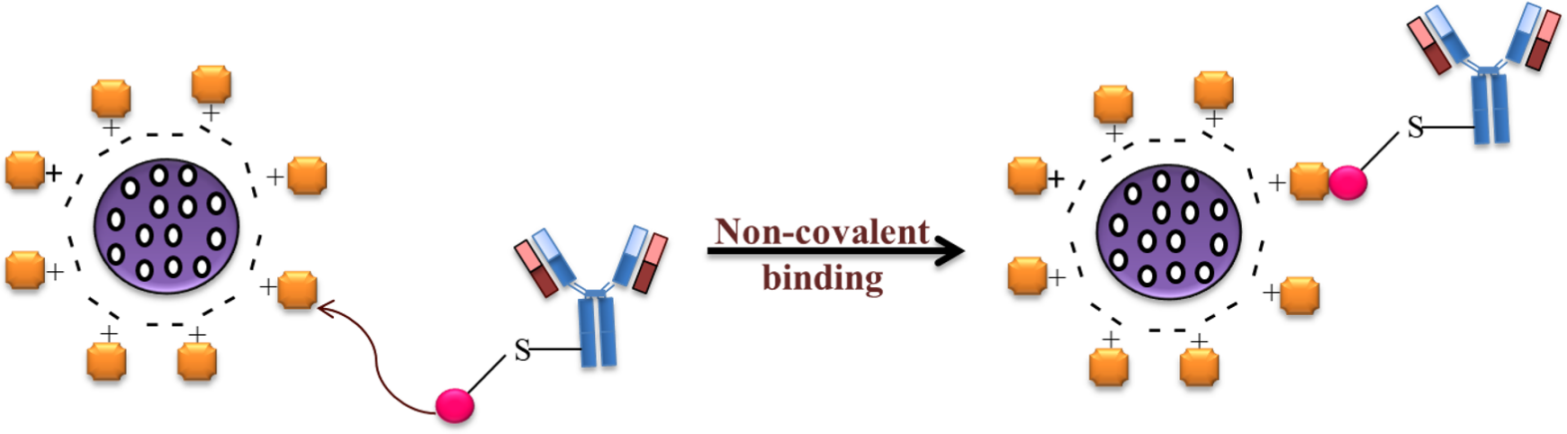
Non-covalent antibody functionalization on NP surfaces using adaptor molecules (such as avidin or streptavidin).

Non-covalent conjugation via adapter molecules ensures the availability of the Fab region of antibodies through the oriented immobilization via the Fc region. The binding of antibodies on nanoparticle surfaces is much stronger, but it is a reversible binding. Biotin–avidin interaction is the most commonly used binding strategy with adapter molecules (Figure 5). It relies on the strong non-covalent interaction between biotin and its binding proteins (avidin and/or its analogues) [63–64]. The bond formed between biotin and avidin forms rapidly and is stable under different conditions of pH, temperature, organic solvents, and denaturing agents. Biotin is an essential water-soluble vitamin for normal cellular function, growth, and cell signaling. Biotin receptors are highly overexpressed in the body; therefore, it is a potential target for a large number of diseases [65]. Avidin is a tetrameric basic glycoprotein with oligosaccharide moieties of three *N*-acetyl glucosamine and four mannose units. The four sugar moieties of avidin bind to biotin with high affinity [66]. Additionally, at physiological pH, the glycosylation of sugar moieties causes non-specific binding of avidin with molecules other than biotin [67]. To avoid this non-specific binding, nonglycosylated and neutral forms of avidin, either natural or recombinant, are used (e.g., streptavidin and neutravidin) [45,64].

Another method to achieve oriented immobilization of antibodies is based upon the use of Fc binding proteins (such as protein-G and protein-A), which have ability to bind precisely with the Fc region of antibodies [68]. Wartlick et al. used biotin/avidin aptamers to efficiently bind and internalize anti-HER2-modified NPs in HER2-overexpressing cells. They demonstrated that ACNPs yield specific tumor targeting as well as enhanced drug delivery. Also, the nanoformulation provided site-specific delivery via receptor-mediated endocytosis and inhibited proliferation and metastasis in tumors that expressed a specific tumor antigen. The use of adaptor molecules to functionalize antibodies resulted in a highly stable conjugation with improved therapeutic efficacy [69].

The covalent binding of amine groups of antibodies with carboxyl groups on the NP surfaces through carbodiimide conjugation is commonly accepted because it does not require a modification of the antibodies. After the conjugation of specific antibodies, the quantification of NP–antibody binding efficacy is an important factor to improve therapeutic efficacy. Researchers are actively working on quantification methods that are specific, rapid, sensitive, simple, and easy to use. The Lowry assay, UV spectroscopy, Bradford assay, and bicinchoninic acid assay are widely used and accepted methods to quantify the concentration of antibodies on NP surfaces in laboratories [70]. Yet, the results produced by these methods are not very precise as each method has its own advantages and disadvantages.

The binding efficacy of antibody-conjugated NPs to overexpressed receptors on target cells is high because of the specific nature of the targeting antibody. ACNPs are expected to accumulate at the target site and to improve the tumor uptake. ACNPs receptor binding efficacies were determined using in vitro studies including cellular uptake and internalization studies [71].

### Multivalent effect of antibody-conjugated NPs

The high surface-to-volume ratio of NPs offers the advantage to manipulate their dimensions and functionalize their surface with multivalent targeting moieties to facilitate targeted delivery in tumor cells [72]. NPs are known to allow multiple ligands to bind on their surface. Multivalent binding is a highly selective interaction because both enthalpy and entropy are involved in the binding thermodynamics [73]. In case of multivalent particles, the entropy loss on binding is less than that of the two molecules in free solution. The multivalent NPs are very specific for the corresponding receptors and, therefore, provide more selectivity for diseased cells. The available evidence suggest that NPs increase the targeting efficacy by providing multiple copies of targeting ligand on the NP surface. This enhances multiple bindings between receptor–ligand pairs [74–75]. Hence, the multivalent effect makes the NPs very selective and provides a high sensitivity for binding on the target surface.

### Multicomponent effect of protein corona on antibody-conjugated NPs

The small hydrodynamic diameter and unique structure of NPs offer increased adsorption and catalytic efficiency. The NP surface potential plays an essential role in the interaction between protein and NPs as opposite charges produce strong interaction and result in high conformational changes [76]. The conjugation of antibodies on nanoparticle surfaces enhances the delivery of drug cargo specifically to disease sites. Once NPs enter the blood stream, they instantly absorb proteins to form a protein corona, which may prominently impede the binding of antibodies to their receptors [77]. There is increasing evidence that NPs conjugated with antibodies, peptides, and aptamers lose the ability to recognize or bind with specific receptors after protein corona formation. Also, NPs that adsorb opsonins, such as immunoglobulins, complement components, and fibrinogen, on their surface are cleared from the body effortlessly by the mononuclear phagocytic system (MPS) [78]. The targeting efficiency of antibody-conjugated NPs is directly affected by their physicochemical properties, including hydrodynamic diameter, surface potential, shape, functional groups, and hydrophobicity. The protein corona reduces the targeted delivery of NPs by disturbing or inhibiting the binding of target molecules to their receptors. Mirshafiee et al. used a copper-free click reaction to decorate fluorescent silica NPs with bicyclononyne. They exposed the NPs to media that mimicked in vitro culture conditions (10% serum) and biological fluids present in vivo (100% serum). They observed an increase in size and a slight decrease in negative charge in serum-containing media, which confirmed protein corona formation. The protein corona establishes a barrier between the ligand and the target, significantly reducing the NP targeting efficiency as compared to bare NPs [79]. Salvati et al. developed transferrin (Tf)-modified ﬂuorescent silica NPs to evaluate the effect of the protein corona on active targeting. Differential centrifugal sedimentation and immunological dot-blot studies revealed that increased serum concentration significantly reduced the binding of Tf-modified NPs to their receptors. They demonstrated that the protein corona significantly reduced the receptor-mediated uptake and internalization of NPs in A549 cells. They clearly indicated a significant disparity between in vivo and in vitro outcomes of NP targeting eﬃcacy in the presence of a protein corona [80]. Su et al. reported that protein corona formation alters the active and passive targeting of cyclic RGD peptides attached on PEGylated NPs. The cellular uptake of NPs with bound proteins was reduced to 26% compared with NPs without bound proteins (ca. 76%). The in vivo results also demonstrated that the targeting efficacy of cyclic RGD peptide-functionalized PEGylated NPs was much smaller when proteins were bound to NPs [81]. Xiao et al. functionalized Tf onto the surface of PEGylated polystyrene NPs to evaluate the effect of the protein corona on blood–brain barrier transcytosis, endocytosis, and intracellular trafficking. They demonstrated that Tf-NPs completely lost their targeting ability after in vitro protein corona formation, while the activity was preserved after in vivo protein corona formation [82].

Protein corona formation on antibody-conjugated NPs can have both negative and beneficial effects. Nayak et al. adsorbed bovine lactoferrin (BLf) onto AgNPs and studied the effect of this protein corona. They observed a higher internalization of BLf-AgNPs than of bare AgNPs, which indicates that the protein corona improved the intracellular efficiency of the NPs. Moreover, the BLf corona also increased the bioavailability of the NPs in THP1 cells and resulted in enhanced humoral immune response. The protein corona formation on Ab-conjugated NPs mediates a more specific and sensitive antibody–antigen interaction [83]. The group of de Puig evaluated the effect of a protein corona on anti-NS1 Ab-conjugated star-shaped gold NPs. They found that the protein corona increased the binding affinity of the NPs to bind to the Zika virus NS1 [84]. In contrast, Dai et al. examined the effect of protein corona formation on the targeting ability of silica-poly(methacrylic acid)-PEG-AntiHER2 NPs. They demonstrated that a protein corona from human serum affects the NPs surface properties by reducing surface charge and availability of conjugated molecules on the NPs, which results in a reduction of targeting capacity from 70% to 7%. However, HAS-incubated NPs exhibited increased interaction between targeting molecule and ligand, boosting the targeting capacity of Afb-conjugated NPs, which further confirmed the multicomponent effect of the protein corona [85]. Based on these findings, it appears that the protein corona significantly alters the properties of the antibody-conjugated NPs in both affirmative and destructive manners.

The protein corona formation results in the reduction or elimination of NP targeting capability by shielding or completely covering relevant functional groups. To block the adhesion of corona proteins on NP surfaces, various strategies have been established using surface barrier layers, such as polymer, protein, or biomimetic coatings, with the ultimate aim to prolong the blood circulation time of NPs. However, some of these strategies inhibit the internalization of NPs by cancer cells, resulting in limited therapeutic effectiveness. Accumulating evidence suggests that protein-repellent coating compounds (zwitterionic compounds) on NP surfaces prevent or minimize the corona formation. Precoating of NPs with specific proteins that increase the adsorption of plasma proteins with intrinsic targeting capacities also improve the targeting ability of the NPs. Moreover, researchers demonstrated that attachment of targeting moieties on the surface of corona-coated NPs increases the targeting capacity. These strategies are useful to shield the effect of protein corona formation and, therefore, improve the biodistribution profile and targeting efficacies of the NPs [86].

### Therapeutic applications of the antibody-conjugated NPs

High metastasis rates, drug resistance, and tumor relapse are the leading challenges in cancer diagnosis and treatment. The commonly used methods for the diagnosis of cancer involve identification of cancer-causing features in cells, such as DNA and RNA mutations, impaired expression of proteins, and changes in confirmation and cell morphology [87]. These methods are very expensive and time-consuming. Additionally, most of the chemotherapeutics are associated with clinical limitations, such as rapid clearance from the blood stream and severe toxic effects [88]. Therefore, researchers have shifted their interest to identify new molecular markers for the rapid detection and treatment of various types of cancers. NPs enhance the drug accumulation at the target site due to their advantages, including higher surface-to-volume ratio, ease of surface modification and functionalization, precise control of structure and size, and enhanced physicochemical features [89–90]. However, NPs can also be restricted by biological barriers; thus, to achieve site-specific delivery, the exterior of NPs is decorated with highly compatible ligands to enhance the receptor-mediated internalization of NPs [91]. The multifunctionalization of NP surfaces with targeting moieties such as antibodies protects the chemotherapeutic agent from enzymatic degradation and improves the internalization into targeted cancer cells. Multifunctionalized NPs improve the tumor targeting ability, boost the body’s antitumor immune response, and decrease the occurrence of systemic inflammatory reactions [92–93]. Multifunctionalization facilitates the specific and selective immunogenic cell death of the cancer cells and also reverses immune suppression [93]. ACNPs combine the advantages of the NPs with high affinity and improve cell penetration through the antibodies [94]. Additionally, these targeted NPs internalize the chemotherapeutics precisely into tumor cells with minimal drug leakage and also provide protection from degradation and elimination [95]. The controlled size and surface charge of NPs avoid the rapid renal clearance of the NPs by the MPS and, ultimately, result in increased blood circulation time [94,96]. The conjugation of targeting moieties on NP surfaces using suitable conjugation methods yields a strong bond and conserves the biological activity of the antibodies. Accumulating literature suggests that ACNPs have great potential and can be effectively used as imaging/therapeutic agents (Table 1).

**Table 1:** Antibody decoration strategies on nanoparticle surfaces and therapeutic applications against various types of cancer.

Nanoparticles	Targeted moiety	Nanoparticle-decorating strategy	Disease	Theranostic applications	Ref.

polymeric NPs	CD133	maleimide chemistry	colorectal cancer	CD133 binding on NP surfaces yielded a highly biocompatible targeted system specifically to eradicate cancer stem cells and suppressed the tumor growth more efficiently.	[97]
polymeric NPs (PEG-PLA)	CK	EDC/NHS	glioblastoma	Surface modification of NPs enhanced the targeted delivery of paclitaxel and provide enhanced antitumor effect in glioblastoma therapy.	[98]
solid lipid NPs	bombesin	EDC/NHS	breast cancer	Targeted NPs significantly improved the anticancer activity by inducing apoptosis in the tumor cells.	[99]
magnetite NPs	NIS	EDC/NHS	differentiated thyroid carcinoma	Anti-NIS antibody-conjugated magnetite NPs acted as diagnostic and therapeutic tool for localizing and treating NIS-expressing tumors.	[100]
solid lipid NPs	aprotinin and melano- transferrin	EDC/NHS	glioblastoma	Surface decoration of NPs enhanced the chemotherapeutic effect of doxorubicin in U87MG cells.	[101]
solid lipid NPs	HER2	streptavidin– biotin interaction	breast cancer	Functionalization of HER2 antibody showed a synergistic effect, increased the internalization, and showed potent antitumor effects on MCF-7 cell lines.	[102]
solid lipid NPs	8314 and anti-epithelial growth factor receptor antibodies	EDC/NHS	glioblastoma	Surface decoration specifically targeted EGFR on U87MG cell and inhibited the growth of glioblastoma.	[103]
polymeric NPs	transzumab	EDC/NHS	breast cancer	Antibody-conjugated polymeric NPs provided site-specific delivery of epirubicin through active targeting and exhibited superior anticancer activity for the treatment.	[104]
bio-reducible NPs	Trop2	EDC/NHS	triple negative breast cancer	Trop2 antibody-conjugated NPs specifically targeted Trop2-expressing TNBC cells and showed improved anticancer efficacy in TNBC-targeted therapy.	[105]
polymeric NPs	Notch 1	EDC/NHS	triple negative breast cancer	Notch 1 conjugation on the surface of polymeric NPs specifically inhibited notch signaling and initiated apoptosis in TNBC cells, simultaneously.	[106]
polymeric NPs	DR5	EDC/NHS	pancreatic cancer	Conjugation of DR-5 increased the extent of apoptosis by downregulating the expression of antiapoptotic protein FADD-like IL-1β-converting enzyme-inhibitory protein (FLIP). Targeted nanoformulation markedly reduced tumor growth with greater efficacy.	[107]
polymeric NPs	DR5	EDC	melanoma	DR5-targeted NPs showed improved antitumor and pro-apoptotic activity.	[108]
polymeric biodegradable NPs	anti-RNEU and anti-CD40 antibodies	adsorption	cancer	Surface modification with two different antibodies enhanced the antitumor response, with complete eradication of the tumor, and also reduce tumor angiogenesis.	[109]
iron-dextran NPs	anti-PD-1 and anti-CTLA-4 antibodies	sulfo-NHS- biotin	cancer	NPs conjugated with two different antibodies concurrently targeted two stages of the cancer immunity cycle, resulting in robust antitumor activity.	[110]
mesoporous silica NPs	CD11b	click chemistry	breast cancer	Targeted NPs reduced the tumor burden significantly and showed enhanced therapeutic eﬃcacy in an orthotopic 4T1 breast tumor model.	[111]
polymeric NPs	herceptin	adsorption	breast cancer	Targeted nanoformulation exhibited great stability, high cell internalization, and stronger cytotoxicity in breast cancer cell lines.	[112]
polymeric NPs	PD-L1	EDC/NHS	gastric cancer	PD-L1-conjugated NPs enhanced the cellular uptake, stimulated the apoptotic signaling pathway. and exhibited improved anticancer efficiency.	[113]
lipidic NPs	CD44	maleimide chemistry	prostate cancer	Surface decoration of NPs resulted in site-specific targeted delivery and showed enhanced therapeutic effect by eliminating cancer-initiating cells.	[114]
polymeric NPs	GPC3	maleimide chemistry	hepatocellular carcinoma	GPC3-targeted NPs exhibited better stability and higher cellular uptake. Also, they significantly inhibited the tumor growth without producing any obvious side effects in HepG2 xenograft mice.	[115]
gold NPs	EGFR	adsorption	cancer	Antibody-conjugated NPs specifically bind to the surface of the cancer cells with 600% greater affinity and enhance the visualization of cancer cells.	[116]
magnetic NPs	VEGF	EDC/NHS	brain Tumor	Conjugation of anti-VEGF antibody to the NP surfaces increased the accumulation in glioma C6 cells and allowed for the selective visualization of intracranial glioma in a rat model.	[117]
superparamagnetic iron oxideNPs	transzumab	glutaralde- hyde crosslinking	breast cancer	Transzumab-conjugated NPs efficiently destroyed 74% of the population of breast cancer cells and acted as a powerful theranostic for HER^+^ breast cancer.	[118]
superparamagnetic iron oxide NPs	CD133	sulfo-SMCC	glioblastoma	Anti-CD133-conjugated NPs were efficiently internalized and used as a fluorescence nanoprobe for molecular imaging of cancer stem cells in glioblastoma.	[119]
gold NPs	anti-Survivin	EDC/NHS chemistry	bladder cancer	Targeted NPs efficiently detected the survivin protein in cancer patients. The detection is highly susceptible in urine by noticing a simple color change from red to gray.	[120]
graphene oxide NPs	Anti-CD59	EDC/NHS chemistry	lung cancer	The antibody-conjugated immune-sensor was highly specific and ultrasensitive for the fast and non-invasive diagnosis of lung cancer.	[121]
magnetic NPs	HER2	EDC/NHS chemistry	cancer cell separation in blood	HER2-conjugated magnetic NPs exhibited a higher magnetic field factor in cancer cells through binding on the cell surface, which resulted in the separation of circulating cancer cells in whole blood.	[122]

In some cases, ACNPs provide opportunities for image-guided therapy with overall theranostic applications. The NP-mediated targeted delivery ultimately provides advanced diagnostic and therapeutic options for early diagnosis and treatment of invasive and metastatic cancers.

Based on these findings, it can be suggested that the ACNPs offer significant advancements in cancer diagnosis and treatment. ACNPs are, therefore, efficacious targeted agents with great theranostic ability, enhancing the overall quality of life in preclinical studies.

Along with enormous progress in preclinical studies including improved intratumor drug delivery, enhanced therapeutic efficacy, and controlled release of chemotherapeutics at tumor sites, researchers evaluated the therapeutic potential of ACNPs in clinical trials. A literature study demonstrated that 13 targeted NPs had been progressed into clinical trials in 2013; however, their therapeutic efficacy in humans has not been proven yet [123]. The available literature portrays a picture of a potential translational gap between preclinical and clinical studies. So far, no ACNPs have been approved by FDA, and there are only very few clinical trials in progress [10,124]. The failure of clinical trials can be attributed to the fact that upon penetration into the tumor vasculature, there are different barriers that have to be crossed to reach and enter into tumor cells [124]. In order to achieve improvement, further investigations are required. Moreover, extensive research is still required in the field of clinical safety of ACNPs to evolve a highly acceptable and beneficial delivery system for cancer theranosis.

### Future perspectives

In cancer therapy, ACNPs have several advantages related to their ability to accumulate at tumor sites. More importantly, surface modification and functionalization of NPs to increase their therapeutic efficacy can be achieved very easily. The recent advancement in targeted delivery introduces the development of surface-modified nanosystems that can be used in screening, detection, and eradication of cancer cells and biomarkers, with great potential in theranostic applications. Despite these advantages, the design and fabrication of targeted NPs for cancer therapy is still very challenging regarding biocompatibility, pharmacokinetics, in vivo targeting efficacy, and cost-effectiveness. The optimization of these variables depends on NP design parameters such as size, shape, charge, composition, preparation method, and surface decorating moiety. The most crucial aspect in the future development of ACNPs will be the design and fabrication of multiple targeting moieties with the ultimate goal of diagnosis and treatment with better efficacy. However, ACNPs have proven their ability to deliver chemotherapeutics specifically to the tumors and are currently the most beneficial targeted conjugated therapy in preclinical studies. However, ACNPs have not been passed phase-III clinical trials yet. It is still very important that researchers understand better the chemistry between NPs and antibodies to develop improved ACNPs. Current knowledge of targeted delivery, tumor vasculature, and interactions of ACNPs in the human body is very limited and, hence, results in failure in clinical trials. To completely exploit the advantages of ACNPs in both preclinical and clinical studies, it is substantial to analyze the gaps between formulation development, selection of appropriate animal models, and clinical safety in humans. Additionally, early detection is one of the most important issues in cancer treatment, as this improves survival rates by approximately five years and also lowers the overall treatment cost. However, it is critical that diagnosis and treatment are extremely accurate, otherwise this would result in misdiagnosis and overtreatment. Unfortunately, the success rate for clinical studies is also very low because of the lack of efficacy. Hence, more advancements are required in the area of targeted delivery to benefit from the advantages of ACNPs in cancer treatment.

## Conclusion

ACNPs are one of the emerging targeted delivery systems. They combine the advantages of NPs and antibodies, such as increased surface to volume-ratio, surface modification and functionalization, and improved cellular uptake and intracellular stability. In cancer chemotherapy, ACNPs have shown immense potential to achieve targeted drug delivery and combination therapy, to overcome drug resistance, to reduce toxicity, to enhance immune response, and to monitor treatment response through theranostic applications. There are numerous preclinical studies demonstrating the above benefits of ACNPs in cancer treatment; however, these advances are not yet clinically transformable because of issues such as lack of tumor specificity and selectivity, antibody selection issues, immune reactions against ACNPs, stability of ACNPs, heterogeneity of tumors, regulatory approvals, scale-up and production, and cost considerations.
